# Association of Choline Acetyltransferase Gene Polymorphisms (SNPs rs868750G/A, rs1880676G/A, rs2177369G/A and rs3810950G/A) with Alzheimer’s Disease Risk: A Meta-Analysis

**DOI:** 10.1371/journal.pone.0159022

**Published:** 2016-07-08

**Authors:** Hai Yuan, Qing Xia, Kang Ling, Xiaotong Wang, Xiumin Wang, Xunping Du

**Affiliations:** 1 Department of Rehabilitation medicine, the Second People's Hospital of Hefei City, 230011 Hefei, Anhui Province, China; 2 Department of Neurology, the Second Affiliated Hospital, Wenzhou Medical University, 325027 Wenzhou, Zhejiang Province, China; 3 Department of Neurology, the Second People's Hospital of Hefei City, 230011 Hefei, Anhui Province, China; Weizmann Institute of Science, ISRAEL

## Abstract

**Background:**

Epidemiological studies have investigated the role of choline acetyltransferase (ChAT) in Alzheimer’s disease (AD). ChAT gene polymorphisms (SNPs rs868750G/A, rs1880676G/A, rs2177369G/A, and rs3810950G/A) may be associated with the risk of AD. In this meta-analysis, we determined the relationship between the four polymorphisms and the risk of AD.

**Methods:**

We searched MEDLINE, EMBASE, and HuGEnet databases for studies linking the four polymorphisms with AD risk. We included 16 articles in our meta-analysis to assess the association between the four polymorphisms and susceptibility to AD by calculating the pooled odds ratios (ORs) and 95% confidence intervals (CIs).

**Results:**

The combined results showed no significant association with rs1880676G/A and rs2177369G/A polymorphisms. The risk of AD (GG+GA versus AA: OR = 0.01, 95%CI = 0.01–0.02, P < 0.05; GG versus GA+AA: OR = 0.85, 95%CI = 0.72–1.00, P = 0.05; GA versus AA: OR = 0.60, 95% CI = 0.37–0.98, P = 0.04) with rs868750G/A polymorphism, or the association of rs3810950G/A polymorphism with AD risk in the overall population (GA versus AA: OR = 0.64, 95% CI = 0.44–0.93, P = 0.02; GG+GA versus AA: OR = 0.62, 95% CI = 0.39–0.97, P = 0.04) or Asian group (GA versus AA: OR = 0.50, 95% CI = 0.32–0.76, P = 0.001, and GG+GA versus AA: OR = 0.46, 95% CI = 0.30–0.09, P = 0.0002) was demonstrated.

**Conclusions:**

Our meta-analysis suggested that rs1880670G/A, and rs2177369 G/A polymorphisms were not risk factors for AD. However, rs3810950G/A, or rs868750G/A genetic polymorphism was a genetic risk factor for the development of AD. The rs3810950G/A polymorphism had a negative effect on the risk of AD for GA or GG+GA genotypes compared with AA in the overall population or Asians.

## Introduction

Alzheimer’s disease (AD), characterized by progressive memory loss and cognitive dysfunction, is the most common neurodegenerative disorder of unknown etiology in the elderly. The neuropathological hallmarks of the disease include deposition of plaques, neurofibrillary tangles, and progressive loss of neurons. The complex interaction between genetic and environmental factors contributes to the development of Alzheimer’s disease (AD). Choline acetyltransferase (ChAT) was found to mediate the pathogenesis of AD [1]. Davies et al reported that the loss of cholinergic neurons or ChAT activity was associated with the development of AD [2]. The deficiency of frontal and hippocampal ChAT activity [3,4] mainly involving cholinergic neurons was associated with AD. Cholinergic activity was correlated with beta-amyloid precursor (APP) metabolism in rats [5–8], which is the neurobiological hallmark of AD [9]. Furthermore, the up-regulation of ChAT activity played an important role in cognitive improvement [10] in hippocampus and frontal cortex of AD.

The ChAT gene, containing 15 exons and the entire sequence of the vesicular ACh transporter, is located on chromosome 10q11.23 [11]. A few SNPs associated with AD risk have been identified [12–14]. A large number of ChAT SNPs were evaluated in AD but the results were equivocal. Mubumbila et al [15] associated SNP 2384G>A polymorphism in the first exon of the ChAT gene with AD risk. A survey of several ChAT SNPs reported the relationship between ChAT and AD risk. However, results from published studies involving Caucasians are contradictory [12,16–18]. Most of these studies were inconclusive and included a relatively small number of cases and controls. Based on the potential role of ChAT in AD pathogenesis, we performed a meta-analysis to investigate the contribution of SNPs including rs868750G/A, rs1880676G/A, rs2177369G/A, and rs3810950G/A to AD risk.

## Materials and Methods

### Search strategy

The electronic databases MEDLINE, EMBASE, and HuGEnet were searched to identify eligible studies without language restriction. The search was only focused on “human” studies. The following Medical Subject Heading (MESH) terms or key words were used: (‘‘choline acetyltransferase” or ‘‘ChAT”) and (‘‘genetic” or ‘‘polymorphism” or ‘‘mutation” or ‘‘genes”) and (‘‘AD” or ‘‘Alzheimer’s disease”). The related reference lists were reviewed to ascertain additional studies.

### Inclusion criteria

Eligible studies met the following criteria: (a) evaluation of 4 SNPs of ChAT gene polymorphisms (SNPs rs868750G/A, rs1880676G/A, rs2177369G/A and rs3810950G/A) and AD risk, (b) clinical diagnosis of AD [19–21], (c) case–control studies, and (d) available data for calculating odds ratios (OR) with 95% confidence interval (CI). The exclusion criteria were: (1) cases with a family history of AD, (2) case reports, editorials, and review articles, and (3) duplicate studies. If a study contained more than one sample, each sample was used for this meta-analysis.

### Data extraction

Two investigators independently reviewed abstracts or full text to identify eligible studies, and extracted data independently. The discrepancies were resolved following discussions. The following information was extracted: the first author, genotyping method, nation and ethnicity of the study population, year of publication, and genotype frequencies in cases and controls.

### Statistical analysis

The strength of the association between AD susceptibility and ChAT gene polymorphism (rs868750 G/A) was evaluated by the odds ratio (OR) and the corresponding 95% confidence interval (CI). Five different ORs were assessed in our analysis: dominant model (GG+GA versus AA), recessive model (GG versus (GA+AA), homozygote comparison (GG versus AA), and heterozygote comparison (GG versus GA, GA versus AA). The same method was used for the other three polymorphisms (rs1880676G/A, rs2177369G/A, and rs3810950G/A).

The test for heterogeneity between studies was performed with Cochran’s Q test. A fixed effects model was used to calculate the pooled OR and 95% CI if the P-value was greater than 0.1, which indicated homogeneity [22]. Otherwise, a random effects model [23] was adopted to combine eligible data with the Review Manager, version 5.3. Significance of the pooled OR was determined by the Z-statistic.

A subgroup analysis was conducted on the basis of ethnicity or APOEε4 carrier status or ethnicity. The sensitivity analysis was performed by the sequential removal of individual studies to explore the stability of the results. The visual Begg’s funnel plot was used to explore publication bias, and Egger’s linear regression test was utilized to quantitatively assess the publication bias with STATA 12.0 software [24]. All genotype distributions of the control population for eligible studies were tested for deviation from Hardy-Weinberg equilibrium using Chi-square test. If the controls were not in accordance with Hardy-Weinberg Equilibrium, the study was excluded for the sensitivity analysis.

## Results

### Study selection

**Fig 1:**
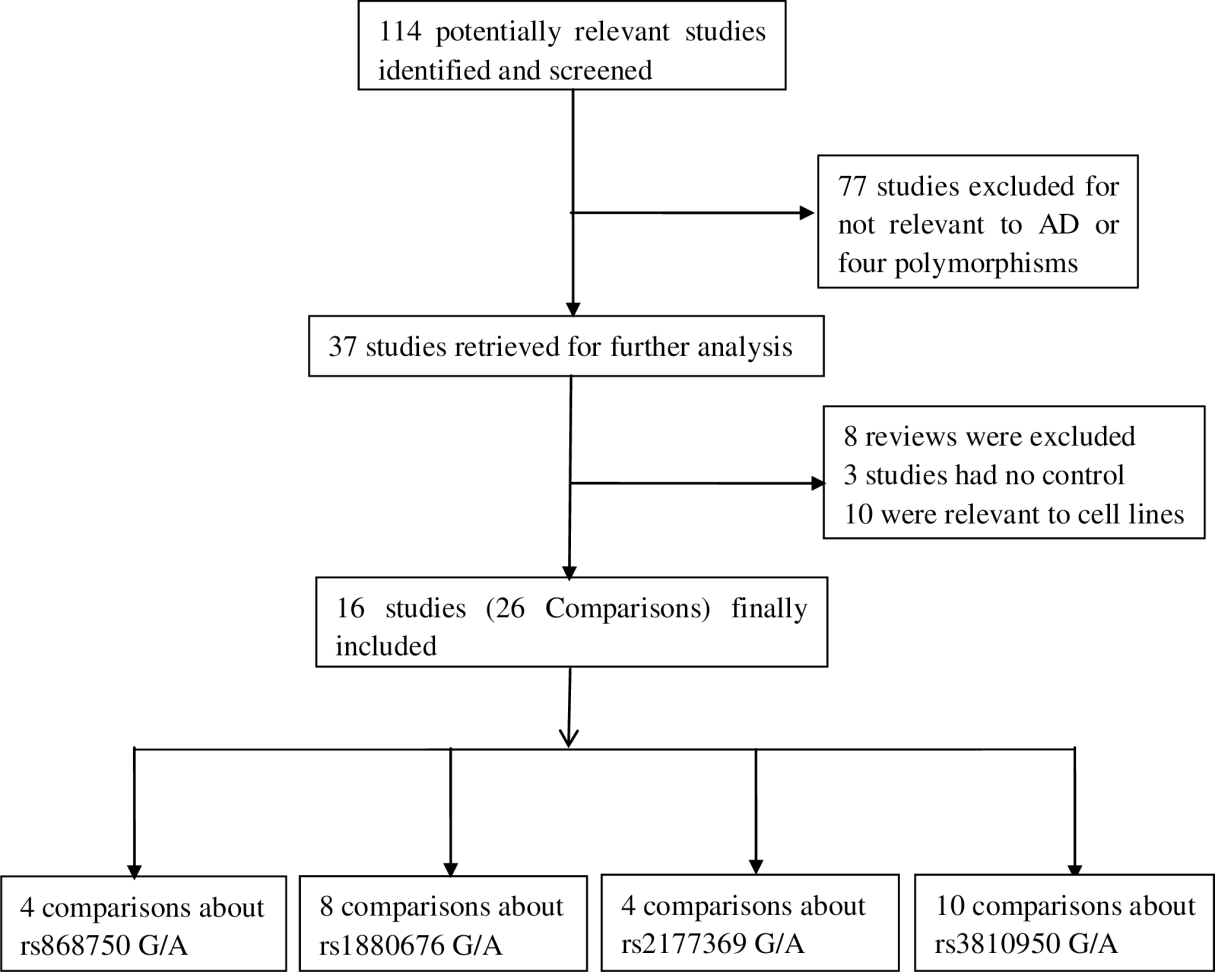
Flow chart of literature search and study selection.

A total of 114 studies were retrieved in the initial search, of which 37 related to ChAT genes and AD were identified. Based on their titles and abstracts, 21 studies were excluded because 8 were reviews, 3 studies had no controls and 10 involved cell lines. Finally, 16 studies or 26 comparisons pertaining to ChAT polymorphisms and AD risk were included. Fig 1 showed the results of the study screen, and the characteristic features of the eligible studies were exhibited in Table 1.

**Table 1:** Characteristics of included studies in this meta-analysis.

Author	Year	Country(Ethnicity)	Genotyping Method	SNP	Diagnosis Criteria	Matching Characteristics	Specimen
Harold	2003	UK (Caucasian)	NP	rs868750 rs1880676 rs3810950	NINCDS-ADRDA	Gender, Age	Blood
Ozturk	2006	USA (Caucasian)	NP	rs868750 rs3810950 rs1880676	NINCDS-ADRDA	Gender, Age	Blood
Ahn Jo	2006	Korea (Asian)	SNaPshot	rs1880676 rs3810950	NINCDS-ADRDA	Age	Blood
Reiman	2007	USA (Caucasian)	NP	rs1880676	NINCDS-ADRDA	Age	Blood
Li	2008	Canada /UK (Caucasian)	PCR	rs1880676	NINCDS-ADRDA	Age	Blood
Giedraitis	2009	Sweden (Caucasian)	PCR	rs1880676	NINCDS-ADRDA	NP	Blood
Scacchi	2008	Italy (Caucasian)	PCR	rs2177369	NINCDS-ADRDA DSM-IV	Gender, Age	Blood
Cook	2004	UK (Caucasian)	NP	rs2177369 rs3810950	NINCDS-ADRDA	Gender, Age	Blood
Piccardi	2007	Italy (Caucasian)	PCR-RFLP	rs2177369	NINCDS-ADRDA DSM-IV	Gender, Age	Blood
Schwarz	2003	Germany (Caucasian)	PCR	rs3810950	NINCDS-ADRDA CERAD	Gender, Age	Blood
Kim	2004	Korea (Asian)	PCR	rs3810950	NINCDS-ADRDA DSM-IV CERAD	Gender, Age	Blood
Tang	2008	China (Asian)	PCR-RFLP	rs3810950	NINCDS-ADRDA DSM-IV	Gender, Age	Blood
Grunblatt	2009	Austria (Caucasian)	PCR	rs3810950	NINCDS-ADRDA DSM-IV CERAD	NP	Blood
Lee	2012	Korea (Asian)	PCR	rs3810950	NINCDS-ADRDA	NP	Blood
Mengel-From	2011	Denmark (Caucasian)	PCR	rs3810950	NINCDS-ADRDA	NP	NP
Mubumbila	2002	France/Germany (Caucasian)	SSCP	rs3810950	NP	Gender, Age	NP

NINCDS-ADRDA, the criteria of the National Institute of Neurological and Communicative Disorders and Stroke (NINCDS) and the Alzheimer’s Disease and Related Disorders Association (ADRDA). CERAD, The Consortium to Establish a Registry for Alzheimer’s disease. Part I. Clinical and neuropsychological assessment of Alzheimer’s disease. NP: Not Provided.

### Study characteristics

Overall, the meta-analysis of included studies comprised 1452/1175 (rs868750G/A), 3341/3013 (rs1880676G/A), 1100/828 (rs2177369G/A) and 3275/3878 (rs3810950G/A) cases/controls(S1 Table). In most studies, the non-demented age- and sex-matched controls were found, diagnoses of definite or probable AD were established according to DSM [19], NINCDS-ADRDA [20] or CERAD [21], genomic DNA was extracted from peripheral tissues according to standard procedure (blood or brain), and genotyping was performed on genomic DNA using a polymerase chain reaction. The 16 studies included 12 European [13–15, 17, 18, 25–31] and 4 Asian [12, 16, 32, 33] populations (Table 1).

### Meta-analysis

**Fig 2:**
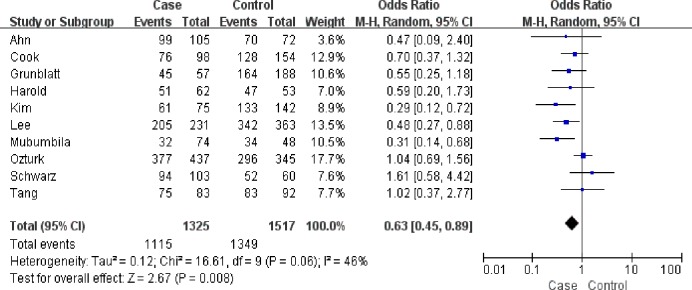
Meta-analysis of the association between the rs3810950G/A gene polymorphism and AD risk (GA versus AA).

**Fig 3:**
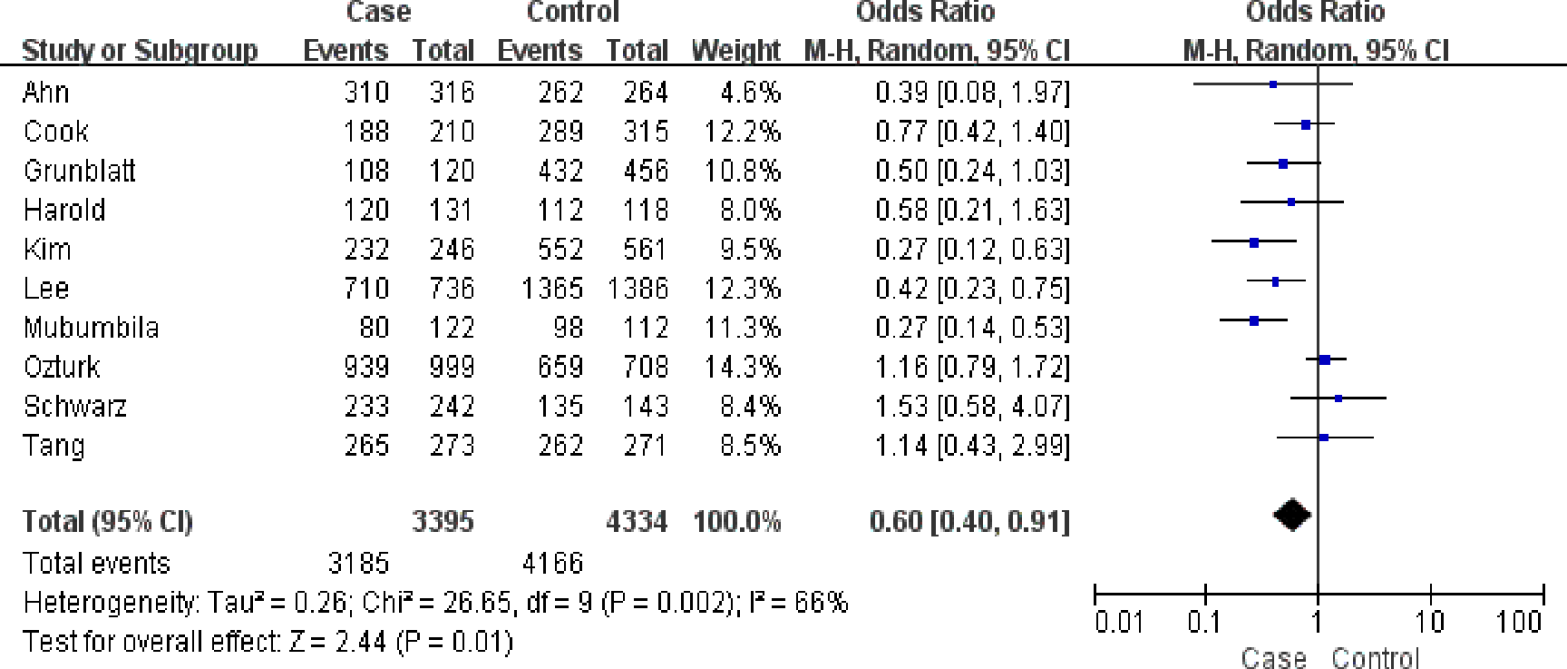
Meta-analysis of the association between the rs3810950G/A gene polymorphism and AD risk (GG+GA versus AA).

A significant association was found between the rs3810950G/A polymorphism and AD risk in the heterozygote model (GA versus AA: OR = 0.64, 95% CI = 0.44–0.93, P = 0.02, P_heterogeneity_ = 0.04, random effects model. Fig 2), the homozygote model (GG versus AA: OR = 0.62, 95% CI = 0.38–1.00, P = 0.05, P_heterogeneity_ = 0.0003, random effects model) and the dominant model (GG+GA versus AA: OR = 0.62, 95% CI = 0.39–0.97, P = 0.04, P_heterogeneity_ = 0.001, random effects model; Fig 3) (Table 2). However, subgroup analysis by APOEε4 carrier status did not reveal significant associations for both APOEε4 carriers (GG+GA versus AA: OR = 0.71, 95% CI = 0.37–1.37, P = 0.31; GG versus GA+AA: OR = 0.84, 95% CI = 0.62–1.14, P = 0.27; GG versus AA: OR = 0.70, 95% CI = 0.36–1.36, P = 0.29; GG versus GA: OR = 0.90, 95% CI = 0.65–1.23, P = 0.49; GA versus AA: OR = 0.75, 95% CI = 0.38–1.50, P = 0.41) and non-APOEε4 carrier populations (GG+GA versus AA: OR = 1.06, 95% CI = 0.65–1.74, P = 0.82; GG versus GA+AA: OR = 0.88, 95% CI = 0.55–1.42, P = 0.60; GG versus AA: OR = 0.78, 95% CI = 0.22–2.73, P = 0.70; GG versus GA: OR = 0.90, 95% CI = 0.59–1.38, P = 0.64; GA versus AA: OR = 1.06, 95% CI = 0.65–1.74, P = 0.82) in three comparisons. In subgroup analysis stratified by ethnicity, the rs3810950G/A polymorphism was associated with AD risk among Asians in the heterozygote and dominant models (GA versus AA: OR = 0.50, 95% CI = 0.32–0.76, P = 0.001; GG+GA versus AA: OR = 0.46, 95% CI = 0.30–0.09, P = 0.0002, respectively), but not in the other models (GG versus GA+AA: OR = 0.80, 95% CI = 0.54–1.19, P = 0.27; GG versus AA: OR = 0.52, 95% CI = 0.21–1.27, P = 0.15; GG versus GA: OR = 0.95, 95% CI = 0.73–1.24, P = 0.69). No association was found in Caucasians (GG+GA versus AA: OR = 0.69, 95% CI = 0.41–1.15, P = 0.16; GG versus GA+AA: OR = 0.89, 95% CI = 0.78–1.01, P = 0.06; GG versus AA: OR = 0.64, 95% CI = 0.39–1.06, P = 0.08; GG versus GA: OR = 0.93, 95% CI = 0.82–1.07, P = 0.32; GA versus AA: OR = 0.71, 95% CI = 0.46–1.09, P = 0.11). The results of subgroup analyses were inconsistent with overall comparisons. Therefore, our meta-analysis demonstrated the association of rs3810950G/A polymorphism and AD risk in the overall analysis or Asian group.

**Table 2:** Meta-analyses of ChAT gene polymorphisms and risk of AD.

SNP	Genotype	No. of comparisons	Test of association			Model	Test of heterogeneity		
			OR	95%CI	*P*		Q	*P*	*I*^*2*^*(%)*
rs868750 G/A	GA vs. AA	4	0.60	0.37,0.98	0.04	F	3.20	0.36	6%
	GG vs. AA	4	0.53	0.33,0.85	0.008	F	5.10	0.16	41%
	GG vs. GA	4	0.89	0.75,1.05	0.16	F	2.52	0.47	0%
	GG vs. GA+AA	4	0.85	0.72,1.00	0.05	F	4.14	0.25	28%
	GG+GA vs. AA	4	0.01	0.01,0.02	<0.05	F	3.31	0.35	9%
rs1880676 G/A	GA vs. AA	8	0.85	0.57,1.25	0.41	R	13.42	0.06	49%
	GG vs. AA	8	0.88	0.57,1.34	0.55	R	16.29	0.02	57%
	GG vs. GA	8	1.06	0.95,1.18	0.30	F	7.04	0.42	1%
	GG vs. GA+AA	8	1.05	0.95,1.16	0.37	F	10.06	0.19	30%
	GG+GA vs. AA	8	0.97	0.78,1.20	0.75	R	15.59	0.03	55%
rs2177369 G/A	GA vs. AA	4	0.82	0.65,1.04	0.09	R	0.47	0.92	0%
	GG vs. AA	4	0.83	0.48,1.44	0.51	R	13.18	0.004	77%
	GG vs. GA	4	1.02	0.63,1.66	0.93	R	11.24	0.01	73%
	GG vs. GA+AA	4	0.93	0.56,1.54	0.78	R	15.77	0.001	81%
	GG+GA vs. AA	4	0.84	0.68,1.03	0.10	F	4.81	0.19	38%
rs3810950 G/A	GA vs. AA	10	0.63	0.45,0.89	0.008	R	16.61	0.06	46%
	GG vs. AA	10	0.60	0.39,0.93	0.02	R	30.45	0.0004	70%
	GG vs. GA	10	0.98	0.88,1.08	0.64	F	11.73	0.23	23%
	GG vs. GA+AA	10	0.89	0.75,1.05	0.17	R	22.68	0.007	60%
	GG+GA vs. AA	10	0.60	0.40,0.91	0.01	R	26.65	0.002	66%

R, random effects model; F, fixed-effects model.

The relationship between rs868750 G/A polymorphism and the risk of AD was found (GG+GA versus AA: OR = 0.01, 95% CI = 0.01–0.02, P < 0.05, P_heterogeneity_ = 0.35, fixed effects model; GG versus GA+AA: OR = 0.85, 95% CI = 0.72–1.00, P = 0.05, P_heterogeneity_ = 0.25, fixed effects model; GG versus AA: OR = 0.53, 95% CI = 0.33–0.85, P = 0.16, P_heterogeneity_ = 0.16, fixed effects model; GG versus GA: OR = 0.89, 95% CI = 0.75–1.05, P = 0.16, P_heterogeneity_ = 0.47, fixed effects model; GA versus AA: OR = 0.60, 95% CI = 0.37–0.98, P = 0.04, P_heterogeneity_ = 0.36, fixed effects model). However, the association with rs1880676G/A, and rs2177369G/A was not investigated. The details were exhibited in Table 2.

### Sensitivity analysis and Publication bias

**Fig 4:**
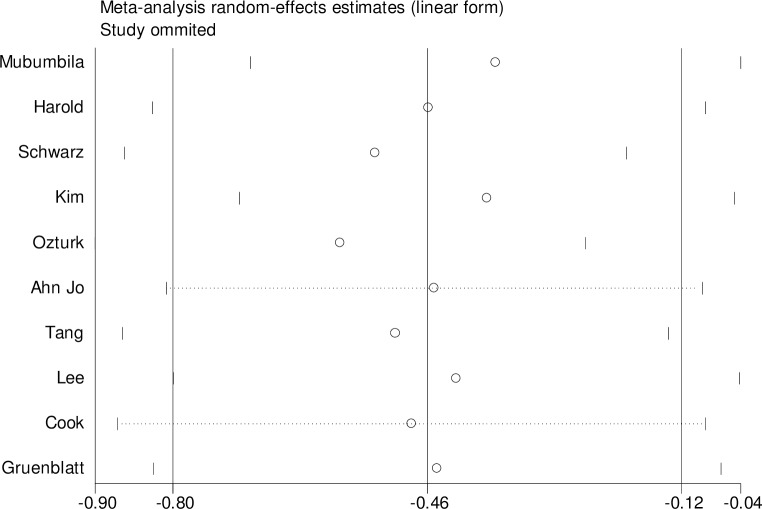
Sensitivity analysis of the relation between the ChAT rs3810950 G/A polymorphism and AD risk (GA versus GG).

**Fig 5:**
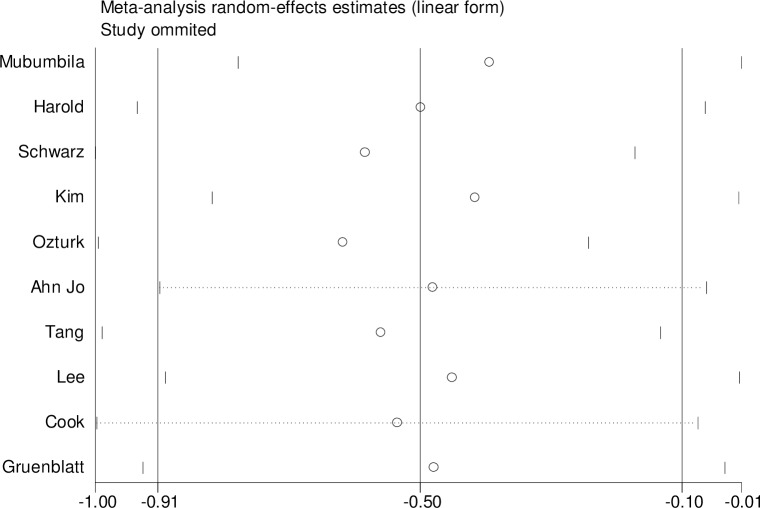
Sensitivity analysis of the correlation between ChAT rs3810950 G/A polymorphism and AD risk (GG+GA versus AA).

**Fig 6:**
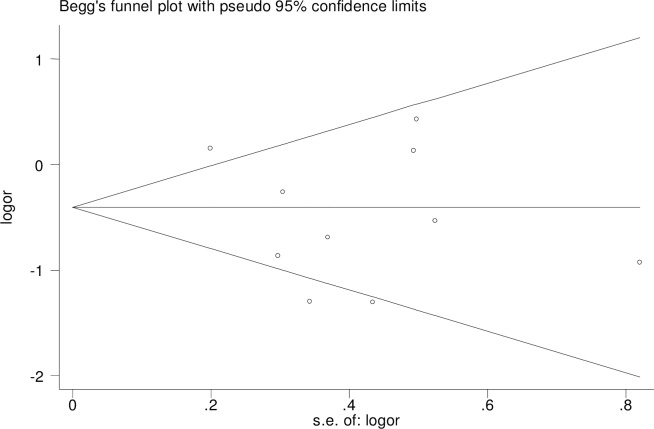
Begg’s funnel plot for rs3810950 G/A gene polymorphism (GG+GA versus AA) and AD risk.

**Fig 7:**
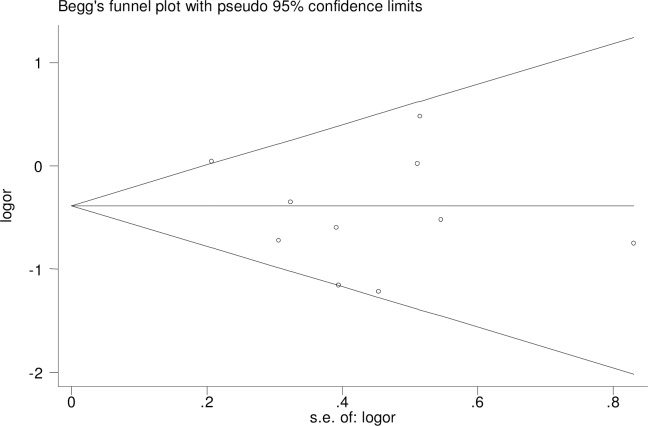
Begg’s funnel plot for rs3810950 G/A gene polymorphism (GA versus AA) and AD risk.

For rs3810950 G/A, after one study [15] was excluded based on the deviation from Hardy–Weinberg equilibrium (χ2 = 0.545, P = 0.011), the two models were unchanged [heterozygote model (GA versus AA: OR = 0.73, 95% CI = 0.58–0.93, P = 0.01 and the dominant model (GG+GA versus AA: OR = 0.67, 95% CI = 0.49–0.99, P = 0.05] indicating that the two overall ORs were stable. Sensitivity analysis was performed by sequential removal of individual studies to reflect the effect of individual data on the pooled ORs. We did not find the effect of any study on the pooled results in the above two models (Figs 4 and 5). We used Begg’s funnel plot and Egger’s linear regression test to determine publication bias. The funnel plot was nearly symmetrical (Figs 6 and 7). The Egger’s linear regression test showed no publication bias (GA versus AA: t = -1.01, P = 0.344, GG+GA versus AA: t = -1.01, P = 0.340).

## Discussion

**Fig 8:**
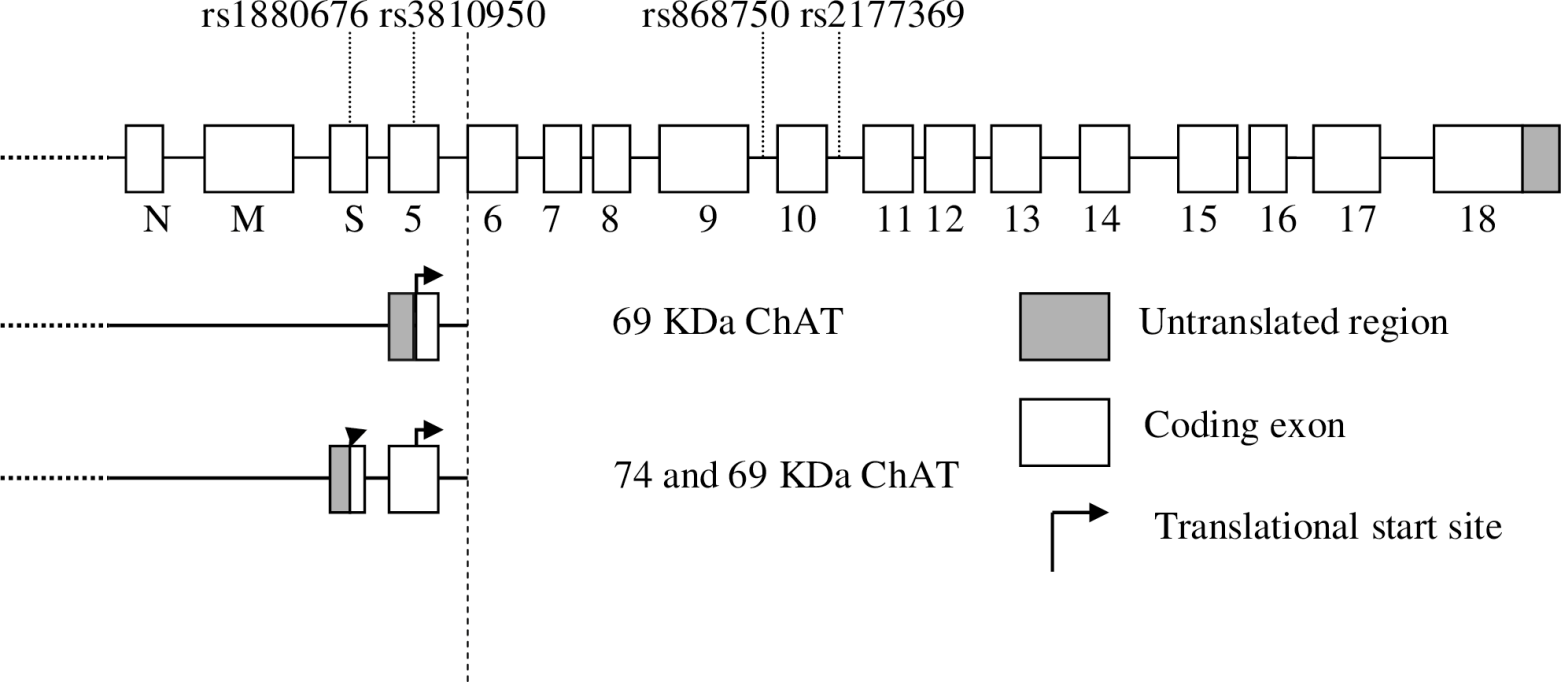
The genomic structure ofthe ChAT. The SNP rs1880676 G/A, rs3810950G/A, rs868750 G/A and rs2177369 G/A is located in exon S, exon 5, intron 9,and intron 10 respectively. The 5 variantencodes the 69-kDa ChATprotein but the S variant encodes a 74-kDaprotein [35–37]. The figure was drawn based on relevant studies [13,15,18,25].

Harold et al identified rs868750 (11604 G/A) using primer extension assay. It was localized to intron 9 or 140 bp downstream of exon 9(Fig 8) [25]. None of the results obtained with the SNP rs868750 G/A polymorphism was significantly associated with AD risk in the three studies from the UK [25]. However, Ozturk et al reported the association of rs868750 G/A polymorphism with AD risk in the USA [13], and also observed that the genetic interaction with APOEε4 carriers played a role. Our results also showed a genetic association with AD susceptibility.A ChAT intron 10 SNP (rs2177369 G/A) was found to be associated with AD risk in a pilot sample of 202 cases and 295 controls (Fig 8) [18]. However, Piccardi et al found no significant difference in the association between rs2177309G/A, and AD risk [29] and was further corroborated by Harold et al [25]. Our meta-analysis also showed no association between rs2177309G/A polymorphism and AD risk. However, in Italy, Scacchi et al showed a significant difference in genotype distribution (χ^2^ = 6.38, P = 0.01), and the ChAT G/G genotype was associated with a higher risk for AD compared with the G/A+A/A genotypes in APOEε4 or non-APOEε4 carriers [14]. Though the biological effect of SNP rs868750 G/A or rs2177369 G/A on AD risk is not explicit, we found a possible association between rs868750G/A but not rs2177309G/A with AD risk. Genetic interaction with APOEε4 carriers might play a role in these associations based on above studies, or the smaller sample size may contribute to inconsistent results. However, the inadequate sample size prevented subgroup analysis in our meta-analysis. Therefore, further investigations with larger and more powerful sample cohorts are needed to reinforce the conclusions.

Rs1880676 (1882G/A, Asp7Asn) was identified by restriction fragment length polymorphism assay and located in exon S (D7N)(Fig 8). If the sequence numbering of CHAT started from the first translational start site in exon S, the amino acids were numbered from the first amino acid of the large 74-kDa isoform of ChAT [25]. However, no rs1880676 G/A was linked to AD pathogenesis. In the UK, no association of genotype (rs1880676 G/A) or allele with the AD compared with controls was reported [25]. In the USA, the lack of association was replicated by Ozturk et al [13]. Ahn Jo et al found that the ChAT genotype distribution between the patients and the controls had no significant difference in Korea [12]. Our results were consistent with most previous studies, and the risk of AD was not associated with rs1880676 G/A polymorphism. However, heterogeneity was found by Cochran’s Q test between the results of individual studies. Subgroup analyses should be conducted to evaluate the effect of heterogeneity on the results of the meta-analysis based on age at onset, ethnicity or geographic distribution of population. However, the original database should be supported by authors. Further investigations with larger sample are needed to confirm or refute these results.

**Fig 9:**
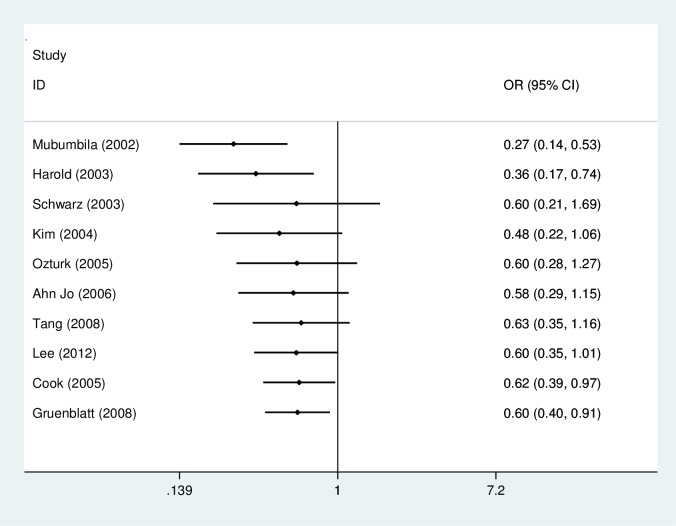
Cumulative meta-analysis of the association between ChAT rs3810950 G/A polymorphism and AD risk of the overalls using a random effects model (GG+GA versus AA).

**Fig 10:**
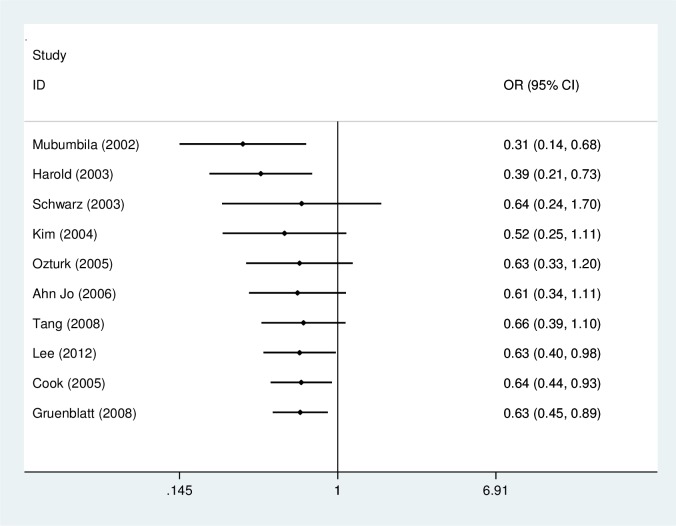
Cumulative meta-analysis ofthe association between the ChAT rs3810950 G/A polymorphism and AD risk of the overalls using a random effects model (GA versus AA).

Mubumbila et al [15] found SNP rs3810950 G/A in the first common coding exon 5 of the ChAT gene, among the combined French and German populations(Fig 8). This G/A transition may contribute to ATG usage resulting in attenuation of translational efficacy of ChAT messenger RNA following substitution of an alanine residue for a threonine (Ala120Thr). However, the biological implication of the association of this SNP with AD is not clear. Kim et al in Korea found that ChAT AA was associated with AD risk in APOEε4 carriers (OR = 43.25, 95% CI = 1.17–9.03) [16]. Tang et al also found the risk for AD associated with ChAT rs3810950 G/A polymorphism in China [32]. Our combined results of overall analysis showed a significant negative association between AD risk and rs3810950 G/A polymorphism, and the lower risk of AD with genotype GA or genotypes GG+GA was found compared with genotype AA. However, Schwarz et al reported that this polymorphism was not associated with AD risk in Germany [17]. Harold et al also failed to replicate the previous associations with AD risk in UK [25]. Several confounding factors might contribute to heterogeneity of meta-analysis or conflicting results. The stratification based on APOEε4 polymorphism or ethnicity might be one such factor. The effect of the rs3810950 A allele on AD risk was consistent with the ApoEε4 allele in a Korean AD population [16]. One study reported that the frequency of the rs3810950 A allele was significantly lower in non-ApoEε4 AD carriers in the USA [13]. A Korean study found an association with GA genotype or GA/AA genotypes in non-ApoEε4 allele carriers with AD [12]. However, stratification ORs of studies showed a significant relationship based on ethnicity. No statistical correlation based on APOEε4 carrier status was found in our meta-analysis and was inconsistent with the overall comparisons. Therefore, the genetic interaction with ethnicity might affect these relationships. A larger sample size is needed to confirm the genetic interaction with APOEε4 polymorphism. When heterogeneity is found between the studies, the results should be interpreted in accordance with cumulative meta-analysis [34]. In our study, the results of cumulative meta-analysis for recessive model GA versus AA or GG+GA versus AA showed stability after the year 2003 (Schwarz) [17] in the overall analysis, respectively (Figs 9 and 10). The recalculated ORs were found stable in two models by excluding single studies from the overall pooled analysis, indicating the validity of our results (Figs 4 and 5). Finally, the funnel plots and Egger’s tests revealed no potential publication bias. The results suggested that the conclusions of the meta-analysis were reliable.

Several limitations should be considered. First, the sample size of included studies was small, which might contribute to the probability of false positive or false negative results. Second, subgroup analysis based on age of onset, samples (blood or brain) or genotyping methods, which might result in selection bias and clinical heterogeneity, was not carried out due to lack of sufficient sample size. Finally, publication bias associated with the exclusion of unpublished studies with negative results was not explored.

Notwithstanding the study limitations, the meta-analysis suggests that the ChAT rs1880670 G/A or rs2177369 G/A polymorphism might not be a risk factor for AD. The rs3810950 G/A or rs 868750G/A genetic polymorphism plays an important role in the development of AD, and the rs3810950 G/A polymorphism showed a negative effect on the risk of AD for GA or GG+GA compared with AA in overall comparisons or Asian populations. Further investigations into the interaction of APOEε4 carrier status or ethnicity with these polymorphisms should be carried out.

## Supporting Information

S1 Table Genotype distribution. (PDF)
