# Supplementary material for: Association of Choline Acetyltransferase Gene Polymorphisms (SNPs rs868750G/A, rs1880676G/A, rs2177369G/A and rs3810950G/A) with Alzheimer’s Disease Risk: A Meta-Analysis
Source: PLoS One. 2016 Jul 8;11(7):e0159022. doi: 10.1371/journal.pone.0159022 (PMC4938620; doi:10.1371/journal.pone.0159022)
Supplement: S1 Table — (PDF) [file pone.0159022.s001.pdf]

S1 Table. Genotype distribution

SNP	Subgroup	Author	Case			Control		
			GG	GA	AA	GG	GA	AA
rs868750 G/A		Harold 1	88	42	5	95	33	3
		Harold 2	129	75	5	130	84	8
		Harold 3	72	39	8	83	31	2
		Ozturk	628	322	39	476	217	13
rs1880676 G/A		Harold 1	34	25	9	49	33	3
		Harold 2	71	56	8	64	62	9
		Harold 3	105	77	12	127	79	3
		Ozturk	563	376	62	369	292	44
		Ahn Jo	211	99	6	193	69	2
		Reiman	478	329	46	303	206	41
		Li	386	256	48	364	275	42
		Giedraitis	54	29	1	222	144	18
rs2177369 G/A		Cook 1	29	85	88	76	124	95
		Cook 2	26	79	74	17	47	36
		Scacchi	167	200	75	61	117	40
		Piccardi	158	44	75	118	40	57
rs3810950 G/A		Mubumbila	48	32	42	64	34	14
		Harold	69	51	11	65	47	6
		Schwarz	139	94	9	83	52	8
		Kim	171	61	14	419	133	9
		Ozturk	562	377	60	363	296	49
		Ahn Jo	211	99	6	192	70	2
		Tang	190	75	8	179	83	9
		Lee	505	205	26	1023	342	21
		Cook	112	76	22	161	128	26
		Gruenblatt	63	45	12	268	164	24
rs3810950 G/A	APOEε4-	Kim	92	37	7	353	118	9
		Ozturk	235	155	18	287	240	39
		Ahn Jo	95	62	2	151	63	2
	APOEε4+	Kim	79	24	7	66	15	0
		Ozturk	321	220	40	76	55	10
		Ahn Jo	116	37	4	41	7	0
